# Dietary and cardio-metabolic risk factors in patients with Obstructive Sleep Apnea: cross-sectional study

**DOI:** 10.7717/peerj.3259

**Published:** 2017-06-21

**Authors:** Marta Stelmach-Mardas, Marcin Mardas, Khalid Iqbal, Magdalena Kostrzewska, Tomasz Piorunek

**Affiliations:** 1 Department of Epidemiology, German Institute of Human Nutrition—Potsdam-Rehbruecke, Nuthetal, Germany; 2 Department of Pediatric Gastroenterology and Metabolic Disorderes, Poznan University of Medical Sciences, Poznan, Poland; 3 Department of Human Nutrition and Hygiene, Poznan University of Life Sciences, Poznan, Poland; 4 Department of Pulmonology, Allergology and Respiratory Oncology, Poznan University of Medical Sciences, Poznan, Poland

**Keywords:** Diet, Biomarkers, Sleep apnea, Obesity, Cardio-metabolic, Risk factor

## Abstract

**Background:**

Little is known about the role of dietary intake in obstructive sleep apnea (OSA), which could prove important in improving clinical outcomes for people with obesity and/or cardiovascular disease within these populations. Reduction in energy intake typically results in weight loss, markedly improving metabolic parameters and ameliorating OSA severity. The aim of this study was to evaluate the association of dietary and cardio-metabolic risk factors with OSA severity.

**Methods:**

This was a cross-sectional study. A total of 75 volunteers at risk of OSA were recruited from 153 patients suffering from sleep disturbance at the Department of Pulmonology, Allergology and Respiratory Oncology at the Poznan University of Medical Sciences. Polysomnography was used for OSA diagnosis. Sleep quality was assessed by the Pittsburgh Sleep Quality Index. Blood pressure, parameters of glucose (fasting glucose, glucose tolerance test) and lipid metabolism (TC, LDL-C, HDL-C, TG) were assessed using routine enzymatic methods. Dietary intake was evaluated by 24-hr dietary recalls and Food Frequency Questionnaire. Ordinal logistic regression models were used for association of background characteristics and dietary intake with OSA severity. All analyses were adjusted for age, sex, BMI, smoking and alcohol intake.

**Results:**

A higher percentage of smokers were observed in patients with mild OSA, while alcohol intake was the highest in severe OSA patients. Approximately 60% of the studied patients were self-reported poor sleepers. Results from ordinal logistic regression models showed that higher intakes of alcohol intake were associated with increased odds of severe OSA; whereas higher HDL-C levels were associated with lower odds (OR 0.01; 95% CI [0.0003–0.55]). Significantly higher odds of high OSA severity were observed in patients with disturbed sleep stages and obstructive sleep apnea. Moreover, the investigation of nutrient intake in relation to OSA severity showed that a higher intake of dietary fiber was associated with decreased OSA severity (OR 0.84; 95% CI [0.71–0.98]).

**Conclusions:**

The severity of OSA is related to higher alcohol consumption and disturbed sleep. The significantly lower dietary fiber intake in patients with severe OSA is of particular importance for dietary consulting in clinical practice, which may positively influence cardiometabolic outcomes.

## Introduction

Obstructive sleep apnea (OSA) is considered a public health problem (Lee et al., 2008) and is associated with an increased risk of diabetes, hypertension and cardiovascular diseases (CVD) (Pepperell, Davies & Stradling, 2002; Wang et al., 2013; Dong, Zhang & Qin, 2013). It has also been suggested that OSA may be independently associated with an increased risk of ischemic heart disease, stroke, arrhythmias and mortality (Lurie, 2011). OSA is characterized by repetitive partial or complete closure of the upper airway during sleep that results in hypoxemia and hypercapnia, is frequently associated with arousals, and leads to an increase in myocardial oxygen demand (Dorasamy, 2007). The sum of the number of apneas and the number of hypopneas per hour is described by the apnea hypopnea index (AHI). It classifies OSA patients into mild (5.0–14.9), moderate (15.0–29.9), and severe (≥30.0 events per hour) (Foster et al., 2009). A number of studies have identified obesity as an important risk factor for OSA (Lee et al., 2008; Gasa et al., 2011). As OSA is diagnosed in approximately 30% of obese patients, metabolic dysfunction often present in obese individuals, including hypertriglyceridemia, lower high-density lipoprotein cholesterol (HDL-C) or hypertension, may play an important role in the increased risk of cardiovascular morbidity and mortality associated with OSA (Gasa et al., 2011; Ahmed & Byrne, 2010).

The unique importance of lifestyle interventions in OSA outcomes has already been emphasized in previous studies (Tuomilehto et al., 2009; Chirinos et al., 2014). Different dietary strategies, based on a reduction in energy intake, have resulted in body weight loss, markedly improving metabolic parameters and ameliorating OSA severity (Gozal, 2015; Vasquez et al., 2008). It has been shown that patients suffering from OSA are characterized by either frequent cravings for carbohydrates or the consumption of greater amounts of total calories derived from protein and fat (saturated fatty acids, trans-fatty acids) (Vasquez et al., 2008).

There is limited literature highlighting diet as an important factor related to AHI, and thus, to severity of OSA. In routine practice of treatment in OSA patients, an adequate dietary intake could result in a step forward in supportive care in these patients. Therefore, we aimed to analyze the association between dietary intake and cardio-metabolic biomarkers with OSA severity in patients with risk of OSA.

## Material and Methods

### Study design and patient’s population

This was a cross-sectional study. One hundred and fifty-three patients were screened at the Department of Pulmonology, Allergology and Respiratory Oncology at the Poznan University of Medical Sciences (the reference center for OSA investigation in the Wielkopolska Region of Poland) for the diagnosis of OSA.

Inclusion criteria were as follows: age >18, signs of OSA (snoring, apnea during sleep, morning tiredness, increased daytime sleepiness etc.), having a habitual diet (no special diet being followed) during the period of examination and willingness to participate in the study. Exclusion criteria included: age <18, pregnancy or lactation, history of cancer (excluding curatively treated with no evidence of disease for five years), severe liver or kidney diseases, and diagnosed CVD including myocardial infarction, stroke, and angina pectoris. Active drug or alcohol abuse, legal incompetence and limited legal competence were additional exclusion criteria.

Seventy-five subjects were enrolled in the study. We set the statistical power at 80% in our sample size. Written informed consent was obtained from all the subjects who agreed to participate in the study. Medical history, comorbidities and concomitant medications were recorded in an electronic database. The Research Ethical Committee at the University of Medical Sciences in Poznan (Poland) approved the study protocol (No. 400/15).

### Anthropometry appraisals, polysomnography and assessment of sleep quality

The anthropometrical parameters included the assessment of body weight and body height with an approximation of 0.1 kg and 0.5 cm (Seca digital scale 763; Seca, Hamburg, Germany). These measurements were used to calculate body mass index (BMI) (Cole, 1990).

We used the laboratory based polysomnography (PSG) with respect to the recommendations of the *American Academy of Sleep Medicine (AASM)* manual (Iber et al., 2007) for OSA diagnosis. The technical details can be found in the paper by Oku & Okada (2008) and Stelmach-Mardas et al. (2016). The total number of apnea and hypopnea episodes related to the total sleep time was described by AHI. The classification of OSA severity was also based on AHI (mild: 5.0–14.9 events per hour, moderate: 15.0–29.9 events per hour, and severe: ≥30.0 events per hour) (Chirinos et al., 2014). The Oxygen Desaturation Index (ODI) was described as clinically significant when the hypopnea was accompanied by a decrease arterial oxygen saturation (SaO2) of 4% compared to SaO2 waking period (Oku & Okada, 2008). The electroencephalogram (EEG) was used to document wakefulness, arousals and sleep stages during the sleep study. The classification of sleep stages was considered as follow: stages N1–N3 (non-REM), and stage R (REM).

We used the Pittsburgh Sleep Quality Index (PSQI) for subjective assessment of sleep quality and disturbances of sleep in study population. Nineteen questions were grouped into the following seven components: sleep latency, sleep duration, subjective sleep quality, habitual sleep efficiency, sleep disturbances, use of sleeping medication and daytime dysfunction. Patients were asked to rate the components from 0 (no difficulty) to 3 (severe difficulty). A global PSQI score was a sum of obtained results, where individuals scoring >5 were considered clinically disturbed or poor sleepers (diagnostic sensitivity of 89.6% and specificity of 86.5%) (Buysse et al., 1989). Data related to lifestyle factors were collected using self-reported questionnaires.

### Assessment of selected cardio-metabolic biomarkers and blood pressure

Blood samples were taken from patients following overnight fasting. The concentrations of serum total cholesterol (TC (mmol/l)), HDL-C (mmol/l), triglycerides (TG (mmol/l)) and glucose (mmol/l) were measured with the use of enzymatic colorimetric methods (Roche Diagnostics Corp., Indianapolis, IN, USA) (Allain et al., 1974; Matsuzaki et al., 1996; Siedel et al., 1993; Sugiuchi et al., 1995; Roeschlau, Bernt & Gruber, 1974). Low-density lipoprotein-cholesterol (LDL-C (mmol/l)) was evaluated according to the formula by Friedewald, Levy & Fredrickson (1972). The obtained results were interpreted according to *the National Health and Nutrition Examination Survey* (Ford et al., 2003) and *the American Diabetes Federation* (American Diabetes Association, 2014), respectively.

The blood pressure (BP) measurements were performed according to the guidelines of *the European Society of Hypertension* (Mancia et al., 2014) with the use of a digital electronic tensiometer (Omron Corp., Kyoto, Japan).

### Nutritional assessment

Dietary intake was assessed by 24-hr dietary recall with an experienced nutritionist checking the data completion (“Dietetyk”, National Institute of Food and Nutrition, Warsaw, Poland). The selected nutrients (expressed in g, mg or µg) and total energy were estimated from the food composition table. In addition, a non-quantitative Food Frequency Questionnaire (FFQ) was used to assess daily, weekly and monthly intake frequency of 109 commonly consumed foods. The FFQ was used to estimate energy intake derived from food consumption frequencies and evaluate whether dietary intake assessment by 24-hr recall could have been affected by systematic error (Table S1).

### Statistical approach

All statistical analyses were conducted using SAS Enterprise version 6.1. (SAS Institute Inc., Cary, NC, USA). Variables with skewed distributions were log-transformed to approximate normal distribution. Missing values were imputed (no. of imputation = 50) using chained equations (Azur et al., 2011) by invoking Proc MI statement in SAS. Fraction missing information and relative efficiency of the imputation are provided in Table S2. A single estimate and standard error for each imputed variable was obtained using Rubin’s rule (Rubin, 1987) by invoking Proc MIANALYZE.

OSA severity categories were defined by AHI cut-offs: AHI <5.0 was categorized as “non-OSA”, 5.0–14.9 as “mild OSA”, 15.0–29.9 as “moderate” and ≥30.0 as “Severe OSA”. Percentages were used to describe categorical variables while mean with standard errors and 95% confidence intervals (95% CI) were used to describe continuous variables. Analysis of variance (ANOVA) and Chi-square were used to test for the differences among OSA categories for continuous and categorical variables, respectively. Differences in background characteristics of the continuous variables across OSA severity categories were described using confidence intervals. OSA severity, with levels ranging from 0-3, was used as an ordinal response variable in assessing its association with lifestyle, cardio-metabolic and dietary factors. Ordinal logistic regression models were used to assess the association of OSA severity categories with basic characteristics (age, sex, smoking status, alcohol consumption), cardio-metabolic (blood glucose, HDL-C, LDL-C, TG, TC), sleep disturbance (central apnea, mixed apnea, obstructive apnea, snoring time) and dietary factors (carbohydrates, protein, fat, dietary fiber, vitamin A, vitamin B12, vitamin B1, vitamin B2, vitamin B6, vitamin C, vitamin D, vitamin E, vitamin PP, potassium, magnesium, iron, zinc, copper). Nutrient densities were used for the association of nutrient intake with OSA severity. Nutrient densities were estimated as nutrient intake/total energy and expressed as g or mg/1,000 kcal. Association of OSA severity levels with cardio-metabolic profile and sleep disturbance was adjusted for age, sex, BMI, smoking and alcohol intake. The association of OSA severity level with dietary factors was further adjusted for energy intake, along with basic lifestyle factors. Results from ordinal logistic regression were presented as odds ratio with 95% confidence interval. In the current analysis, we considered a double-sided *p*-value less than 0.05 for significance.

## Results

Study baseline characteristics are presented in Table 1. Patients with normal AHI (no OSA) were younger than those with OSA. Mean BMI values in all AHI groups exceeded 30 kg/m^2^. However, patients with OSA (mild, moderate, and severe) had higher BMI as compared to those with no OSA. A high proportion of smokers were observed in the group of patients with a mild OSA (AHI 5-14.9). Comparatively higher intakes of alcohol were observed in patients with moderate (57.28 g/day; 95% CI [55.83–58.73]) and severe (95.9 g/day; 95% CI [93.61–98.19]) OSA. According to the PSQI, approximately 60% of the studied individuals described their quality of sleep as poor. There were no differences in education, marital status, residence and financial status across AHI patient groups. Due to small sample sizes mean differences in smoking categories could not be estimated.

**Table 1:** Basic characteristics of studied patients classified according to the categories of Apnea/Hypopnea Index representing severity of Obstructive Sleep Apnea (*n* = 75).

Analyzed variables^a^	Apnea/Hypopnea Index (events/hour)				*p*-value^b^
	<5.0 (Normal)	5.0–14.9 (Mild OSA)	15.0–29.9 (Moderate OSA)	≥30.0 (Severe OSA)
Patients n(%)	18.00 (24.00)	11.00 (14.70)	21.00 (28.00)	25.00 (33.30)	
Age (year)	54.48 ± 3.14 (53.78, 55.18)	57.11 ± 2.68 (56.5, 57.72)	57.41 ± 2.49 (56.84, 57.98)	58.8 ± 2.54 (58.23, 59.37)	0.65
Sex (%): Female	61.10	27.30	33.30	44.00	0.23
BMI (kg/m^2^)	30.61 ± 1.51 (30.27, 30.95)	31.5 ± 2.44 (30.96, 32.04)	34.0 ± 1.26 (33.71, 34.29)	32.0 ± 1.23 (31.73, 32.27)	0.32
Smokers (%)	17.10	57.10	16.00	7.40	0.01
Alcohol intake (g/day)	48.5 ± 6.28 (46.94, 50.06)	47.11 ± 5.31 (45.91, 48.31)	57.28 ± 6.42 (55.83, 58.73)	95.9 ± 10.09 (93.61, 98.19)	<0.001
Poor sleep: score > 5					
Poor sleep	61.10	55.40	62.00	60.00	0.98
Education (%)					
Primary school	16.70	0.00	20.00	19.60	0.14
High school	44.40	90.90	45.40	65.40	
University degree	38.90	9.10	34.60	15.00	
Marital Status (%)					
Single	5.60	9.10	6.90	21.80	0.27
Married	77.80	81.80	78.60	61.20	
Divorced	5.60	9.10	14.60	16.80	
Widowed	11.10	0.00	0.00	0.20	
Residence (%)					
<10,000 residents	24.20	18.20	37.00	32.70	0.86
10–50,000 residents	23.70	18.20	26.80	23.80	
50–100,000 residents	11.90	9.10	15.50	7.00	
>100,000 residents	40.20	54.50	20.80	36.60	
Financial Status (%)					
Poor	0.00	19.30	20.60	29.20	0.26
Good	88.90	70.50	74.10	70.10	
Very good	11.10	10.20	5.30	0.70	

**Notes.**

a Data presented as percentage or means with standard errors with 95% confidence interval.

b Values from one-way analysis of variance for continuous variables and Chi-square test for categorical variables. PSQI †, Pittsburgh Sleep Quality Index.

Cardio-metabolic markers, dietary intake and factors describing sleep architecture across categories of OSA severity are described in Table 2. The concentration of TG was higher in patients with severe OSA (2.10 mg/dl; 95% CI [2.05–2.15]) as compared to non-OSA subjects (1.50 mg/dl; 95% CI [1.45–1.55]), whereas HDL-C was higher in non-OSA (1.4 mmol/dl; 95% CI [1.38–1.42]) as compared to severe OSA patients (1.1 mmol/dl; 95% CI [1.08–1.12]). Patients from this group also had the highest values of systolic (131.30 mmHG; 95% CI [130.60–132.00]) and diastolic (79.70 mmHG; 95% CI [79.25–80.15]) blood pressure. Significant differences were also observed in the NREM phase of sleep (stage of sleep 1 and 2) across the analyzed groups of patients (non-OSA vs severe-OSA). The highest values of ODI were also observed in the severe OSA group.

**Table 2:** Comparison of cardio-metabolic, dietary intake and factors describing sleep architecture across categories of Apnea/Hypopnea Index representing obstructive sleep apnea (*n* = 75).

Analyzed variables^a^	Apnea/Hypopnea Index (events/hour)				*p*-value^i^
	<5.0 (Normal)	5.0–14.9 (Mild OSA)	15.0–29.9 (Moderate OSA)	≥30.0 (Severe OSA)
Cardio-metabolic risk factors					
TC (mmol/dl)^b^	5.30 ± 1.40 (5.10, 5.50)	5.5 ± 1.40 (5.09, 5.91)	4.7 ± 1.30 (4.52, 4.88)	5.2 ± 1.40 (4.93, 5.47)	0.35
TG (mmol/dl)^c^	1.50 ± 0.20 (1.45, 1.55)	1.40 ± 0.20 (1.35, 1.45)	1.30 ± 0.10 (1.28, 1.32)	2.10 ± 0.20 (2.05, 2.15)	0.03
LDL-C (mmol/dl)^d^	3.40 ± 0.20 (3.35, 3.45)	3.70 ± 0.50 (3.59, 3.81)	3.00 ± 0.20 (2.95, 3.05)	3.50 ± 0.20 (3.45, 3.55)	0.35
HDL-C (mmol/dl)^e^	1.40 ± 0.10 (1.38, 1.42)	1.30 ± 0.10 (1.28, 1.32)	1.30 ± 0.10 (1.28, 1.32)	1.10 ± 0.10 (1.08, 1.12)	0.01
Glucose tolerance test (mmol/dl)	7.10 ± 0.80 (6.92, 7.28)	6.50 ± 10 (6.27, 6.73)	7.80 ± 1.10 (7.55, 8.05)	8.90 ± 10 (8.67, 9.13)	0.23
Fasting glucose level (mmol/dl)	5.50 ± 0.20 (5.45, 5.55)	5.30 ± 0.20 (5.25, 5.35)	5.80 ± 0.40 (5.71, 5.89)	5.90 ± 0.30 (5.83, 5.97)	0.40
Blood pressure (mmHg)					
Systolic	124.00 ± 3.60 (123.19, 124.81)	122.20 ± 6.50 (120.73, 123.67)	133.50 ± 3.60 (132.69, 134.31)	131.30 ± 3.10 (130.60, 132.00)	0.04
Diastolic	78.70 ± 2.40 (78.16, 79.24)	71.00 ± 3.60 (70.19, 71.81)	82.20 ± 2.50 (81.63, 82.77)	79.70 ± 20 (79.25, 80.15)	0.04
Polysomnography					
ODI (events per hours)^f^	3.80 ± 1.10 (3.55, 4.05)	8.40 ± 1.30 (8.11, 8.69)	24.90 ± 1.60 (24.54, 25.26)	54.80 ± 4.00 (53.89, 55.71)	<0.01
Snoring time (%)	17.10 ± 5.20 (15.92, 18.28)	34.80 ± 9.00 (32.76, 36.84)	34.70 ± 6.80 (33.16, 36.24)	25.25 ± 5.90 (23.91, 26.59)	0.17
NREM Phase (%)^g^ Stage of sleep N1	7.40 ± 0.80 (7.22, 7.58)	8.70 ± 1.90 (8.27, 9.13)	23.10 ± 5.80 (21.79, 24.41)	11.50 ± 2.40 (10.96, 12.04)	0.01
Stage of sleep N2	10.40 ± 1.70 (10.02, 10.78)	15.70 ± 3.20 (14.98, 16.42)	14.40 ± 1.60 (14.04, 14.76)	23.10 ± 3.50 (22.31, 23.89)	0.01
Stage of sleep N3	31.10 ± 3.10 (30.4, 31.8)	33.30 ± 4.10 (32.37, 34.23)	27.40 ± 3.70 (26.56, 28.24)	28.60 ± 3.70 (27.76, 29.44)	0.30
REM Phase (%)^h^ Stage of sleep R	27.60 ± 4.40 (26.60, 28.60)	26.80 ± 4.10 (25.87, 27.73)	16.80 ± 3.50 (16.01, 17.59)	17.40 ± 2.70 (16.79, 18.01)	0.08
Nutrients					
Energy intake (kcal)	1800.40 ± 142.10 (1768.24, 1832.56)	1245.20 ± 117.80 (1218.54, 1271.86)	1136.10 ± 114.00 (1110.30, 1161.90)	1546.10 ± 89.40 (1525.87, 1566.33)	<0.01
Carbohydrates (g)	228.60 ± 21.90 (223.64, 233.56)	153.50 ± 18.30 (149.36, 157.64)	141.50 ± 14.20 (138.29, 144.71)	188.60 ± 13.20 (185.61, 191.59)	<0.01
Protein (g)	74.30 ± 3.90 (73.42, 75.18)	63.70 ± 7.80 (61.93, 65.47)	53.60 ± 6.30 (52.17, 55.03)	72.90 ± 5.60 (71.63, 74.17)	0.04
Fat (g)	74.50 ± 7.60 (72.78, 76.22)	48.90 ± 5.60 (47.63, 50.17)	45.00 ± 6.90 (43.44, 46.56)	62.70 ± 4.80 (61.61, 63.79)	0.01
Dietary fibre (g)	21.60 ± 2.00 (21.15, 22.05)	16.80 ± 2.10 (16.32, 17.28)	13.10 ± 1.60 (12.74, 13.46)	17.10 ± 1.30 (16.81, 17.39)	0.01
Vitamin A (ug)	904.20 ± 163.80 (867.13, 941.27)	601.10 ± 129.20 (571.86, 630.34)	655.50 ± 101.80 (632.46, 678.54)	905.90 ± 159.50 (869.8, 942)	0.33
Vitamin B12 (ug)	3.00 ± 0.40 (2.91, 3.09)	2.50 ± 0.20 (2.45, 2.55)	2.80 ± 1.00 (2.57, 3.03)	4.30 ± 0.80 (4.12, 4.48)	0.36
Vitamin B1(mg)	1.20 ± 0.10 (1.18, 1.22)	1.00 ± 0.10 (0.98, 1.02)	1.00 ± 0.20 (0.95, 1.05)	1.20 ± 0.10 (1.18, 1.22)	0.55
Vitamin B6 (mg)	1.70 ± 0.10 (1.68, 1.72)	1.50 ± 0.20 (1.45, 1.55)	1.30 ± 0.20 (1.25, 1.35)	1.70 ± 0.20 (1.65, 1.75)	0.25
Vitamin C (mg)	48.30 ± 7.20 (46.67, 49.93)	47.00 ± 13.10 (44.04, 49.96)	51.50 ± 14.30 (48.26, 54.74)	38.00 ± 6.00 (36.64, 39.36)	0.76
Vitamin D (ug)	2.90 ± 0.50 (2.79, 3.01)	2.20 ± 0.50 (2.09, 2.31)	3.20 ± 1.40 (2.88, 3.52)	5.40 ± 1.90 (4.97, 5.83)	0.46
Vitamin E (mg)	9.60 ± 1.10 (9.35, 9.85)	9.10 ± 1.90 (8.67, 9.53)	5.40 ± 0.70 (5.24, 5.56)	7.90 ± 0.80 (7.72, 8.08)	0.02
Vitamin PP (mg)	14.50 ± 1.40 (14.18, 14.82)	14.50 ± 2.40 (13.96, 15.04)	13.00 ± 1.90 (12.57, 13.43)	15.10 ± 1.60 (14.74, 15.46)	0.84
Sodium (mg)	2207.00 ± 17.10 (2016.23, 2397.77)	1310.00 ± 20.10 (1177.33, 1442.67)	1353.00 ± 17.30 (1144.49, 1561.51)	1792.00 ± 18.10 (1618.16, 1965.84)	<0.001
Potassium (mg)	2582.00 ± 165.10 (2544.63, 2619.37)	2306.00 ± 256.20 (2248.02, 2363.98)	2143.00 ± 234.80 (2089.86, 2196.14)	2568.00 ± 180.80 (2527.08, 2608.92)	0.35
Calcium (mg)	499.60 ± 46.80 (489.01, 510.19)	385.50 ± 67.40 (370.25, 400.75)	335.40 ± 48.70 (324.38, 346.42)	537.70 ± 75.80 (520.54, 554.86)	0.08
Copper (ug)	1.00 ± 0.10 (0.98, 1.02)	0.90 ± 0.10 (0.88, 0.92)	0.80 ± 0.10 (0.78, 0.82)	0.90 ± 0.10 (0.88, 0.92)	0.31
Iron (mg)	10.20 ± 0.80 (10.02,10.38)	8.30 ± 0.70 (8.14,8.46)	6.80 ± 0.70 (6.64,6.96)	8.90 ± 0.60 (8.76,9.04)	0.01
Zinc (mg)	10.00 ± 0.70 (9.84,10.16)	9.50 ± 0.90 (9.30,9.70)	7.00 ± 1.00 (6.77,7.23)	9.10 ± 0.60 (8.96,9.24)	0.06
Magnesium (mg)	236.50 ± 16.90 (232.68,240.32)	198.10 ± 20.40 (193.48,202.72)	172.00 ± 18.3 (167.86,176.14)	248.00 ± 20.4 (243.38,252.62)	0.02

**Notes.**

a Data presented as means with standard errors and 95% confidence interval.

b TC, total cholesterol.

c TG-triglycerides.

d LDL-C, low-density lipoprotein cholesterol.

e HDL-C, high-density lipoprotein cholesterol.

f ODI, Oxygen Desaturation Index.

g NREM, no rapid eye movement.

h REM, rapid eye movement expressed as percentage of total sleep.

i Values from one-way analysis of variance for continuous variables and Chi-square test for categorical variables. PSQI †, Pittsburgh Sleep Quality Index.

Analysis of nutrient intake showed a reduced energy intake in severe-OSA patients (1,546.1 kcal; 95% CI [1,525.87–1,566.33]) as compared to non-OSA patients (1,800.40 kcal; 95% CI [1,768.24–1,832.56]). Similarly, lower intake of carbohydrates and fats were observed in severe OSA as compared to non-OSA patients. Likewise, severe-OSA patients had significantly lower intake of fiber (17.1 g/day; 95% CI [16.81–17.39]) and Vitamin E (7.9 g/day; 95% CI [7.72–8.08]) as compared to non-OSA patients (21.6 g/day; 95% CI [21.15–22.05]) and 9.6 g/day; 95% CI [9.35–9.85], respectively. Similar results were also observed for magnesium. Intake of selected nutrients was also found to differ from guideline recommendations (Jarosz & Bułhak-Jachymczyk, 2008). Using the estimation of energy intake from the FFQ, we did not detect any systematic error in food intake assessment (Table S1).

**Table 3:** Association (Odds ratio with 95% confidence intervals) of lifestyle, cardio-metabolic and dietary factors with severity of obstructive sleep apnea (*n* = 75).

Variables	ODDS ratio	95% confidence interval	*P*-value
**Raw model**			
Age	1.04	(0.99, 1.08)	0.10
Sex	1.26	(0.79, 2.03)	0.33
BMI (kg/m^2^)	1.6	(0.88, 2.91)	0.12
Smoking status	1.04	(0.96, 1.13)	0.36
Alcohol consumption	1.03	(1.01, 1.04)	<.0001
**Cardiometabolic risk factors**^a^			
Glc (mmol/dl)^b^	5.95	(0.15, 242.35)	0.34
SBP (mmHg)^g^	1.03	(0.99, 1.09)	0.17
LDL-C (mmol/dl)^c^	0.32	(0.03, 3.05)	0.32
HDL-C (mmol/dl)^d^	0.01	(0.0003, 0.55)	0.02
TC (mmHg)^e^	4.09	(0.41, 40.43)	0.23
TG (mmHg)^f^	1.18	(0.14, 9.79)	0.88
**NREM Phase**^a^			
Stage of sleep N1	8.24	(2.12, 32.05)	0.003
Stage of sleep N2	7.31	(1.92, 27.78)	0.004
Stage of sleep N3	7.64	(1.36,42.84)	0.02
**REM Phase**^a^			
Stage of sleep R	1.0	(0.95, 1.05)	0.49
**Types of sleep apnea**^a^			
Central apnea	1.1	(0.52, 2.34)	0.51
Mixed apnea	1.47	(0.69, 3.16)	0.06
Obstructive apnea	5.17	(2.8, 9.55)	<.0001
Snoring time	1.14	(0.73, 1.77)	0.84
**Nutrients**^a^^,^^h^			
Carbohydrates (g)	1.02	(0.98, 1.07)	0.07
Protein (g)	0.99	(0.96,1.03)	0.80
Fat (g)	1.00	(0.98, 1.03)	0.17
Dietary fibre (g)	0.84	(0.71, 0.98)	<0.01
Vitamin A (ug)	1.00	(1, 1.01)	0.50
Vitamin B12 (ug)	1.00	(0.99, 1.01)	0.57
Vitamin B1 (mg)	1.01	(1, 1.03)	0.01
Vitamin B2 (mg)	1.01	(0.98, 1.04)	1.00
Vitamin B6 (mg)	0.8	(0.04, 18.12)	0.07
Vitamin C (mg)	1.00	(0.99, 1.01)	0.24
Vitamin D (mg)	1.00	(0.99, 1.01)	0.57
Vitamin E (mg)	1.00	(0.99, 1.01)	0.42
Vitamin PP (mg)	1.03	(0.82, 1.3)	0.32
Potassium (mg)	1.01	(0.98, 1.04)	0.58
Magnesium (mg)	1.00	(0.98, 1.02)	0.15
Iron (mg)	1.05	(0.51, 2.16)	0.68
Zinc (mg)	0.81	(0.49, 1.34)	0.30
Copper (mg)	1.18	(0.07, 19.63)	0.33

**Notes.**

a Adjusted for age, sex, BMI, smoking and alcohol intake.

b Glc, fasting glucose.

c LDL-C, low-density lipoprotein cholesterol.

d HDL-C, high-density lipoprotein cholesterol.

e TG, triglycerides.

f TC, total cholesterol.

g SBP, systolic blood pressure.

h Further adjustment for energy intake Nutrients are presented as intakes per 1,000 Kcal.

Results from ordinal logistic regression models assessing association of lifestyle factors with OSA categories of severity showed that a higher intake of alcohol was significantly associated with increased odds of being in an OSA category of higher severity as compared to non-OSA (Odds Ratio (OR) 1.03; 95% CI [1.01–1.04]). Assessment of cardiometabolic parameters in relation to OSA categories of severity showed that patients with higher HDL-C levels had significantly lower odds (OR 0.01; 95% CI [0.0003–0.55]) of OSA severity. Association between sleep stages with OSA severity also showed significantly increased odds of high severity in patients with disturbed sleep stages. Furthermore, OSA severity was also related with obstructive sleep apnea in these patients (OR 5.17; 95% CI [2.8–9.55]). Finally, the investigation of nutrient intake in relation to OSA severity showed that higher intakes of dietary fiber was associated with decreased OSA severity (OR 0.84; 95% CI [0.71–0.98]), whereas higher intakes of Vitamin B1 was associated with increased odds (OR 1.01; 95% CI [1–1.03]) of OSA severity (Table 3).

## Discussion

In this study we present the association between severity of OSA, defined according to AHI categories, with selected lifestyle, dietary and cardio-metabolic factors. Alcohol consumption and poor sleep profile were associated with increased OSA severity, whereas higher levels of HDL-C and intake of dietary fiber were associated with lower OSA severity.

Most of the patients suffering from OSA were self-reported poor sleepers (82%) according to the PSQI (Lau et al., 2013), which was also confirmed in our study. Consequently, poor sleep status in OSA patients is usually associated with fatigue and may result in a decrease in physical activity and a compensatory increase in caloric intake leading to weight gain (Stelmach-Mardas et al., 2016). In the current study, mean BMI values in all AHI groups exceeded 30 kg/m^2^, although the mean energy intake did not meet the recommended value (Jarosz & Bułhak-Jachymczyk, 2008). Unfortunately, obesity is very often related to underreporting of food intake by patients, frequently observed in clinical practice and well described in the literature (Johnson, Goran & Poehlman, 1994; Tooze et al., 2004; Stice & Durant, 2014; Nadeem et al., 2014). Furthermore, our patients reported intake from the last 24-hours prior to clinic entry (single intake), so health status and psychological factors related to stress of medical examination in the upcoming day could have also influenced self-reported dietary intake. Nevertheless, 24-hr dietary recall is considered to be a more reliable and unbiased method than FFQ for dietary assessment in the dietary field (Eck, Klesges & Klesges, 1996). Here, we used FFQ frequency information to estimate energy intake using portion sizes as usual consumption amounts to assess whether 24-hr recall intake could have been affected by systematic error. From the estimation of energy intake, we did not detect any systematic error in food intake assessment. In the presented statistical model, we have shown selected lifestyle factors (alcohol consumption) and nutrients (dietary fiber) are associated with OSA severity. Smith et al. (2014) also showed a negative association between liking food rich in dietary fiber and OSA severity and a positive association with high fat food, which could be explained by high levels of ghrelin and leptin resistance. Regarding lipid profile parameters, we observed that the severe OSA group was characterized by lower concentrations of HDL-C. Unfortunately, even future treatment of OSA patients using continuous positive airway pressure (CPAP) may not positively influence the lipid profile, as might be expected (Goris, Meijer & Westerterp, 2001). As Xu et al. (2014) pointed out based on their meta-analysis, CPAP does not alter TG, LDL-C or HDL-C levels, suggesting that it may have no clinically important effect on lipid metabolism. Furthermore, the lipid profile studied in patients also partially reflects the dietary habits, which should be taken under consideration by health professionals to support OSA patients with dietary consulting. Additionally, decreasing HDL-C and increasing TG can be observed with increasing severity of obesity. It was suggested that the AHI can be considered as one of the main indicators of HDL dysfunction (Xu et al., 2014). Moreover, the formation of reactive oxygen species (ROS) is related to the resistive breathing causes respiratory muscle and furthermore may lead to damage of cardiac tissue (Zuo et al., 2012; Zuo et al., 2013). Various nutrient diets can affect ROS levels in OSA patients. Generally, diet can be assessed by using composite evidence-based scores that comprise multiple nutrients and food groups—for instance the Alternate Healthy Eating Index (AHEI) (Mattei et al., 2016). An association between higher AHEI (which reflects better diet quality, based on current evidence) and lower risk of diabetes, CVD, and Metabolic Syndrome has been confirmed in different studies (McCullough et al., 2002; Fung et al., 2007; Belin et al., 2011; Akbaraly et al., 2010; Chiuve et al., 2012). This can be explained by the inclusion of specific foods and nutrients, such as legumes (lower risk), sugar-sweetened beverages, and red or processed meats (higher risk) in the AHEI, which may play a role in the development of chronic diseases (Chiuve et al., 2012). Behavioural changes in food desire under sleep deprivation can also explained the positive association between B1 intake and OSA due to the more often selected food sources of this vitamin (Greer, Goldstein & Walker, 2013). According to data from The National Health and Nutrition Examination Survey III (NHANES III), the prevalence of hypertension can reach up to 42% in men at a BMI of >30 kg/m^2^ and up to 38% in women (Brown et al., 2000). A 10 kg higher body weight is associated with a 3.0 mm Hg higher systolic and a 2.3 mm Hg higher diastolic blood pressure, leading to increased risk of CVD (Reisin, 1986). Furthermore, according to the American Heart Association, obesity can be associated with alterations in hemodynamics, as an increase in oxygen demand produced by excess adipose tissue (∼1.5 mL/kg per minute) requires an increase in cardiac output related to a dysregulation of peripheral vascular resistance (Poirier et al., 2006). In the present study, we have examined mostly obese patients, in which the differences in diastolic blood pressure were highly pronounced across the AHI groups.

The monitored number of desaturation events was also the highest in the group of patients with a high AHI value, which shows that ODI is a very good predictor of AHI. It has already been confirmed by Chung et al. (2012) that ODI can be used as a sensitive and specific tool in clinical practice to detect undiagnosed sleep-disordered breathing. Relief of apneas prevents deep NREM sleep. However, apneas are more commonly observed in stages I and II of NREM and in REM sleep (Chung et al., 2012). It has also been shown that age and sex differences play a role in breathing, especially when compared with breathing during REM sleep (Subramanian, Mattewal & Surani, 2013). However, in our study, sex and age distribution was similar across AHI groups, only showing differences close to significance in the R stage of sleep. Nevertheless, as suggested by Liu et. al Liu et al. (2011) and Subramanian, Mattewal & Surani (2013), patients with either a higher NREM-AHI than REM-AHI or a higher REM-AHI than NREM-AHI should be considered as a part of the spectrum of OSA, rather than a specific clinical entity.

### Limitation

Our study has some limitations. This is a cross-sectional study and hence causality cannot be inferred. We did not apply an objective measure of physical activity, therefore, energy expenditure was not assessed. However, obese patients with OSA are usually characterized by sedentary to moderate physical activity (Stelmach-Mardas et al., 2016). Furthermore, single 24-hour dietary recalls were collected, therefore dietary intake may not reflect fully habitual dietary intake. Finally, due to the high misclassification of sodium (reported either only as part of consumed products or an additionally added amount to meals), this nutrient was excluded from final analysis.

## Conclusions

The severity of OSA is related to high alcohol consumption, lower HDL-C and disturbed sleep. The significantly lower dietary fiber intake in patients with higher AHI is of high importance for dietary consultation in clinical practice, which may positively influence cardiometabolic outcomes.

##  Supplemental Information

10.7717/peerj.3259/supp-1 Data S1 Raw data Nutritional data, raw data for energy intake estimation

10.7717/peerj.3259/supp-2 Table S1 Energy estimation from FFQ

10.7717/peerj.3259/supp-3 Table S2 Variance information

## Additional Information and Declarations

### Data Availability

The following information was supplied regarding data availability: The raw data has been supplied as a Supplementary File.
