# Supplementary material for: Dietary and cardio-metabolic risk factors in patients with Obstructive Sleep Apnea: cross-sectional study
Source: PeerJ. 2017 Jun 21;5:e3259. doi: 10.7717/peerj.3259 (PMC5482261; doi:10.7717/peerj.3259)
Supplement: Data S1 — Nutritional data, raw data for energy intake estimation [file peerj-05-3259-s001.pdf]

An assessment of energy intake from food frequency questionnaire based on the portion size con

Ice Cream	Milk/Milk Products	Cheese	Refined Bread	Whole Bread	Breakfast Cereals	Rice/Pasta	Poultry
0,03	0,07	1,21	4,21	0,21	0,03	0,03	0,29
0,29	2,00	3,00	0,00	1,03	0,14	0,03	0,43
0,03	0,14	3,14	2,36	0,00	0,00	0,29	0,43
0,00	0,00	0,00	0,00	0,00	0,00	0,00	0,00
0,29	0,36	3,07	1,43	0,36	0,03	0,39	0,29
0,00	0,00	0,50	0,24	0,50	0,07	0,07	0,03
0,00	0,36	2,00	0,00	0,00	0,00	0,00	0,00
1,00	0,36	6,07	0,36	0,36	0,00	0,29	0,43
0,00	0,00	0,00	0,00	0,00	0,00	0,00	0,00
0,29	0,14	2,57	0,39	0,39	0,00	0,39	0,43
0,00	0,00	0,32	0,14	1,00	0,36	0,36	0,43
0,29	0,14	2,53	0,18	1,00	0,00	0,50	0,43
0,00	2,00	0,00	0,00	0,00	0,00	0,00	0,00
0,03	1,03	1,57	1,24	1,36	0,03	0,29	0,43
0,03	1,36	1,53	2,14	0,00	0,00	0,50	0,00
0,03	2,14	1,57	0,10	1,03	0,03	0,18	0,00
1,00	0,14	4,29	1,03	0,00	0,00	0,00	0,29
0,03	0,03	1,39	2,10	0,03	0,00	0,29	1,00
0,03	0,03	1,18	0,35	0,03	0,00	0,71	0,43
0,03	0,36	1,50	1,29	2,00	0,00	0,29	0,29
0,03	2,00	1,07	0,03	0,39	0,36	0,18	0,43
0,03	1,00	1,64	0,64	0,71	1,00	0,29	0,29
0,29	0,36	2,10	1,00	0,29	0,00	0,71	0,29
0,43	0,29	4,07	0,36	0,36	0,00	0,14	0,29
0,29	0,29	3,03	2,03	0,03	0,00	0,07	0,43
0,43	1,36	3,68	3,00	1,00	0,36	0,14	0,29
0,03	0,50	1,42	0,71	0,36	0,00	0,07	0,29
0,03	0,50	1,00	0,00	2,00	0,00	0,18	0,29
0,29	0,71	2,39	2,07	2,00	0,03	0,29	0,43
0,00	0,00	0,43	0,36	0,36	0,00	0,36	0,29
0,00	0,00	0,36	0,00	0,36	0,00	0,14	0,43
0,43	0,50	3,07	0,03	0,18	0,00	0,29	0,03
1,00	2,00	5,18	2,03	0,00	0,03	0,18	0,00
0,29	0,36	3,00	0,14	0,50	0,36	0,50	0,29
0,00	0,00	0,00	0,00	0,00	0,00	0,00	0,00
0,43	2,00	3,32	0,00	1,36	0,03	0,18	0,29
0,29	1,00	2,14	0,00	2,00	0,00	0,29	0,29
0,29	0,14	2,13	1,71	0,00	0,36	0,29	0,43
0,43	1,36	5,07	2,53	0,03	0,03	0,71	0,43
0,43	1,14	3,32	3,00	1,36	1,00	0,29	0,29
0,43	0,14	3,00	1,00	0,00	0,00	0,07	0,29
0,43	0,50	3,39	0,18	1,14	0,00	0,18	0,43
0,29	0,71	2,03	0,50	0,00	0,14	0,29	0,43
0,03	0,39	1,07	2,03	0,00	0,00	0,07	0,03
2,00	2,00	5,00	6,00	2,00	0,00	2,00	0,43
0,29	0,18	2,57	0,46	0,07	0,00	0,07	0,43

0,03	2,00	3,43	2,42	0,50	0,07	0,29	0,43
2,00	0,14	5,50	4,03	0,00	0,00	0,18	0,43
0,03	0,00	1,64	1,32	1,03	0,14	0,14	0,43
0,00	1,03	0,03	2,00	1,00	0,00	0,50	0,29
0,29	1,36	2,03	0,36	0,14	0,00	0,03	0,29
0,03	0,18	1,39	4,00	0,00	0,03	0,14	1,00
0,43	1,14	3,00	1,14	1,00	0,00	0,29	0,43
0,03	1,00	1,57	0,10	2,03	0,00	0,29	0,29
0,03	2,00	1,36	0,00	1,00	0,36	0,71	0,29
0,03	0,36	1,50	0,36	0,36	0,00	1,03	0,03
0,03	1,03	1,13	0,32	2,36	0,00	0,18	0,43
0,03	1,00	1,36	0,07	1,36	0,36	0,03	0,43
0,00	0,36	0,29	0,29	0,71	0,00	0,00	0,29
0,00	0,39	0,46	3,03	1,00	0,03	0,29	0,29
0,03	0,39	2,03	2,75	2,00	0,14	0,29	0,43
0,00	0,03	0,18	0,00	0,71	0,36	0,00	0,43
0,43	0,36	4,07	1,00	1,00	0,14	0,29	0,29
0,43	3,00	4,43	1,18	0,29	0,29	0,50	0,29
0,03	0,14	1,00	0,00	0,00	0,03	0,00	0,29
0,03	1,14	1,57	0,07	2,14	1,00	0,07	0,29
0,00	1,14	1,14	2,00	0,00	0,00	0,71	0,43
0,00	1,00	0,00	2,00	0,00	0,36	0,14	0,29
0,00	0,00	0,00	1,36	1,00	0,00	0,50	0,43
0,00	0,00	0,14	2,00	0,00	0,00	0,00	0,43
0,43	2,00	4,14	2,57	0,18	0,07	0,14	0,43
0,43	0,71	3,42	0,42	0,71	0,03	0,29	0,29
0,03	0,71	1,18	0,00	1,36	0,36	0,29	0,43
0,43	4,00	4,43	0,24	0,71	0,00	0,00	0,43
0,43	1,36	4,50	3,00	0,00	0,00	0,50	0,43

ısumption - raw data

Sausages	Red Meat	Fish	Eggs	Vegetables	Green Leafy Vegetables	Margarine	Fruits
0,00	0,39	0,18	0,00	1,63	0,18	0,03	1,86
0,43	0,86	0,71	0,29	3,57	1,43	0,00	5,39
0,29	0,39	0,18	0,29	0,85	0,29	0,00	0,63
0,00	0,00	0,00	0,00	0,00	0,00	0,00	0,00
0,43	0,53	0,29	0,29	2,18	0,36	0,71	2,28
0,29	1,03	0,29	0,29	1,52	0,21	1,00	1,66
0,00	0,00	0,00	0,00	0,00	0,00	0,00	0,00
0,00	0,50	0,50	0,43	2,57	0,10	0,00	4,32
0,00	0,00	0,00	0,00	0,00	0,00	0,00	0,00
1,00	0,36	0,18	0,29	3,68	0,53	0,00	6,42
0,00	0,14	0,00	0,29	1,96	0,36	0,00	3,35
0,03	0,50	0,29	0,29	2,07	0,57	0,29	1,67
0,00	0,00	0,00	0,00	0,00	0,00	0,00	5,43
0,29	0,53	0,07	0,29	5,74	0,10	2,00	2,62
0,43	0,00	0,14	0,43	1,50	0,07	0,43	1,02
0,03	0,14	0,29	0,43	3,68	1,03	0,00	5,06
0,29	0,07	0,03	0,00	1,18	0,71	0,00	1,13
0,43	0,71	0,07	0,29	1,41	0,10	2,03	0,69
0,03	0,36	0,18	0,00	5,46	0,50	1,00	5,00
1,00	0,29	0,29	0,00	3,92	0,39	1,00	2,17
0,03	0,03	0,18	0,29	2,78	1,18	0,43	1,09
0,29	0,32	0,18	0,00	1,75	0,57	2,43	2,45
0,43	0,21	0,14	0,00	2,06	0,13	0,00	0,62
0,00	0,14	0,29	0,43	0,78	0,36	2,00	0,00
0,29	0,14	0,00	0,29	1,99	0,07	1,00	0,34
0,00	0,71	0,18	0,43	1,39	0,64	0,00	2,24
0,29	0,50	0,18	0,29	0,31	0,00	0,00	0,46
0,43	0,00	0,03	0,29	2,89	0,21	2,00	2,68
0,29	0,29	0,07	0,29	1,39	0,21	1,00	4,18
0,43	0,29	0,14	0,29	1,57	0,71	0,00	3,36
0,00	0,14	0,29	0,43	1,07	0,03	1,00	1,24
1,00	0,64	0,18	0,00	3,34	0,35	0,43	1,87
2,00	0,39	0,18	0,00	1,68	0,18	0,00	2,02
0,43	0,71	0,39	0,29	1,71	0,29	0,00	3,00
0,00	0,00	0,00	0,00	0,00	0,00	0,00	0,00
0,29	0,14	0,14	0,43	3,46	0,35	0,00	3,09
0,00	0,14	0,14	0,00	3,79	0,71	0,00	2,50
0,00	0,18	0,18	0,29	0,86	0,32	0,00	4,46
0,29	0,39	0,07	0,43	3,29	0,39	0,32	1,92
0,29	0,36	0,18	0,29	1,82	0,42	0,00	1,90
0,03	0,14	0,71	0,00	2,64	0,71	0,00	16,00
0,03	0,29	0,03	0,29	2,20	1,07	1,43	2,89
0,00	0,10	0,71	0,00	8,14	2,71	0,00	3,96
0,03	0,07	0,00	0,03	0,30	0,10	0,00	0,20
0,43	0,00	0,00	0,43	18,00	4,36	0,43	11,93
0,00	0,53	0,07	0,00	0,44	0,10	0,00	0,30

1,00	0,42	0,29	0,00	3,35	0,42	2,00	1,74
1,00	0,36	0,18	0,29	1,39	0,18	0,86	2,03
0,00	0,32	0,18	1,00	1,79	0,10	0,00	1,70
0,00	0,07	0,50	0,03	5,57	1,50	2,00	2,31
0,43	0,03	0,07	0,00	1,64	0,00	0,00	1,36
0,29	0,18	0,00	0,29	1,92	0,36	0,00	1,09
0,29	0,14	0,50	0,03	5,79	1,18	1,00	2,36
0,29	0,18	0,07	0,03	0,99	0,18	0,32	0,40
0,29	0,14	0,29	0,00	2,14	0,71	0,00	0,71
0,00	0,36	0,00	0,43	1,53	0,03	1,00	2,99
0,43	0,39	1,14	0,03	1,42	1,10	0,00	2,01
0,29	0,71	0,29	0,43	3,64	0,75	1,00	7,12
0,00	0,00	0,00	0,00	0,00	0,00	1,00	0,00
0,29	1,03	0,29	0,29	3,57	0,36	0,03	4,55
0,29	0,39	0,18	0,29	1,46	0,03	0,46	1,78
0,00	0,14	1,36	0,29	3,25	0,42	0,00	3,03
0,29	0,29	0,00	0,43	2,50	1,43	0,00	3,29
0,00	0,32	0,29	1,00	1,31	0,21	0,00	2,95
0,00	0,14	0,14	0,29	2,79	0,36	0,00	2,68
0,43	0,18	0,18	0,43	2,38	0,46	0,71	2,94
2,00	1,00	0,07	1,00	2,60	0,10	2,00	0,71
0,43	0,36	0,00	0,29	2,64	1,00	2,00	1,14
0,00	1,00	0,00	0,00	3,43	0,64	0,29	7,43
0,43	0,36	0,00	0,29	1,21	0,36	2,00	3,13
0,43	0,86	0,18	0,29	3,46	1,07	0,29	2,77
0,03	0,18	0,18	0,43	4,43	0,46	1,00	2,99
0,00	0,50	0,29	0,29	1,43	1,00	1,00	2,03
0,43	0,39	0,71	0,43	2,06	0,46	0,00	1,54
2,00	0,39	0,29	0,43	2,86	0,36	0,00	4,31

Berries	Butter	Lard/Animal Fat	Plant Oil	Quark/Cream	Sweets	Nuts	Salty Beverages
0,00	2,00	0,03	1,03	0,71	0,32	0,29	0,00
2,43	0,00	0,03	1,50	0,36	0,86	0,29	0,00
0,21	2,00	0,00	0,14	0,50	1,64	0,00	0,00
0,00	0,00	0,00	0,00	0,00	0,00	0,00	0,00
0,68	0,43	0,29	1,29	0,71	0,43	0,29	0,00
2,07	2,00	0,03	0,36	0,29	1,21	0,03	0,00
0,00	0,00	0,00	0,00	1,00	0,00	0,00	0,00
1,86	2,00	0,03	1,10	0,71	0,21	0,00	0,00
0,00	0,00	0,00	0,00	0,00	0,00	0,00	0,00
1,43	2,00	0,03	0,03	0,29	3,71	0,43	3,00
1,50	0,29	0,03	0,79	1,00	0,18	0,29	0,00
0,39	1,00	0,03	0,29	0,29	0,14	0,29	0,00
0,43	2,00	0,00	4,00	3,00	1,00	0,00	0,00
0,17	0,29	0,00	0,03	1,03	1,68	0,03	5,00
0,24	0,43	0,43	0,39	0,71	0,00	0,00	0,00
2,24	2,00	0,03	1,86	0,71	0,21	0,03	0,00
0,18	0,43	0,03	3,00	0,18	0,24	0,43	1,00
0,24	0,00	0,03	0,07	0,07	3,32	0,00	2,00
1,25	2,00	0,29	0,00	0,71	0,68	0,03	0,00
2,00	2,00	0,00	1,14	0,71	0,46	0,03	0,00
1,21	0,00	0,00	0,71	0,36	0,00	0,03	0,00
0,60	0,00	0,00	0,57	0,71	0,43	0,00	3,00
0,18	0,43	0,00	0,18	1,00	0,50	0,03	2,00
0,00	0,00	0,00	0,00	0,36	1,00	0,00	0,00
0,42	1,00	0,00	0,00	0,03	0,24	0,03	1,00
0,75	2,00	0,03	0,32	0,71	0,93	0,03	0,00
0,64	1,00	0,03	0,14	0,14	0,29	0,03	0,00
0,00	0,00	0,00	0,18	0,14	0,18	0,29	1,00
1,14	0,43	0,03	0,00	0,29	1,03	0,03	1,00
1,00	2,00	0,00	1,00	0,14	1,00	0,00	0,00
0,07	0,00	0,03	0,00	0,36	0,00	0,00	0,00
0,28	0,43	0,00	0,71	0,29	0,03	0,00	0,00
0,24	2,00	0,03	1,03	1,14	0,89	0,03	1,00
1,00	1,00	0,00	0,36	2,00	0,03	0,00	0,00
0,00	0,00	0,00	0,00	0,00	0,00	0,00	0,00
0,36	0,43	0,00	0,36	1,03	0,57	0,43	0,00
1,57	0,00	0,00	0,43	0,36	0,00	0,00	3,00
2,71	2,00	0,00	0,79	0,14	0,29	0,43	0,00
0,71	2,00	0,03	0,39	0,50	0,93	0,29	2,00
0,17	2,00	0,00	0,39	1,14	2,86	0,03	1,00
5,00	1,00	0,00	1,21	2,03	0,32	0,00	0,00
0,39	1,00	0,00	0,14	0,29	0,53	0,00	1,00
2,53	1,00	0,00	2,03	1,03	0,35	0,43	0,00
0,03	0,00	0,00	0,14	0,07	0,13	0,00	0,00
1,79	0,00	0,43	0,36	2,36	0,43	0,00	0,00
0,32	2,00	0,00	0,14	0,18	0,13	0,00	0,00

0,71	0,29	0,00	0,36	0,50	0,71	0,29	3,00
0,50	0,00	0,29	0,71	0,03	1,57	0,00	3,00
0,93	2,00	0,00	0,00	0,29	0,53	0,00	0,00
0,29	0,03	0,00	0,93	0,03	0,64	0,29	0,00
0,14	2,00	0,03	0,00	1,36	0,50	0,00	0,00
0,35	2,00	0,03	0,07	0,50	0,93	0,29	2,00
0,29	0,43	0,00	0,36	0,00	1,29	0,00	0,00
0,13	0,03	0,03	0,13	0,18	0,71	0,03	0,00
0,29	1,00	0,00	0,14	0,36	0,14	0,29	0,00
0,71	0,43	0,00	0,14	0,50	0,10	0,03	0,00
0,17	0,43	0,00	0,68	0,14	0,24	0,43	1,00
4,10	0,03	0,00	3,10	0,36	0,07	0,29	0,00
0,00	0,00	0,43	0,00	0,50	0,00	0,00	4,00
0,17	0,03	0,03	0,07	1,03	0,57	0,29	0,00
0,24	0,03	0,03	0,36	0,03	1,03	0,00	0,00
0,14	0,00	0,00	0,50	0,03	0,79	2,00	2,00
3,43	1,00	0,03	0,00	0,50	0,29	0,29	0,00
0,75	2,00	0,00	1,79	0,39	0,71	0,29	2,00
3,00	0,00	0,00	0,14	0,03	0,14	0,00	0,00
0,17	0,29	0,03	1,14	0,50	0,71	1,00	2,00
0,24	0,03	0,43	0,36	0,50	0,46	0,03	1,00
1,00	0,00	0,00	0,00	1,00	0,00	0,00	0,00
3,00	0,43	1,00	0,43	0,00	2,00	0,00	0,00
0,21	1,00	0,00	1,00	0,14	0,03	0,00	0,00
1,42	2,00	0,00	0,46	0,50	1,14	0,29	1,00
4,21	1,00	0,03	0,60	0,36	0,10	0,29	0,00
0,89	0,43	0,00	0,36	0,50	0,14	0,29	0,00
0,17	2,00	0,00	3,00	1,36	0,82	0,43	2,00
0,86	2,00	0,29	1,14	0,36	3,00	0,03	1,00

Confectionary (Waffles)	Tea	Kakao	Fruit and Vegetables Juice	Fast Food	Beer	Vodka/Whisky
0,00	1,14	0,00	0,00	0,00	3,00	0,18
0,00	0,00	0,00	0,00	0,36	0,00	0,00
0,00	2,50	0,00	1,00	0,29	2,00	0,03
0,00	0,00	0,00	0,00	0,00	0,00	0,00
2,00	1,07	2,03	4,00	0,00	4,00	0,00
1,00	1,03	1,00	0,03	0,10	0,00	0,03
0,00	0,00	0,00	0,00	0,00	0,00	0,00
0,00	3,00	2,00	0,00	0,00	2,00	0,03
0,00	0,00	0,00	0,00	0,00	0,00	0,00
3,00	2,00	1,03	2,00	0,14	0,00	0,00
0,00	1,36	2,00	0,00	0,07	1,00	0,00
0,00	1,07	0,39	0,71	0,10	1,00	0,00
0,00	6,00	2,00	0,00	0,14	0,00	0,00
2,00	2,07	0,03	0,07	0,57	2,00	0,03
0,00	0,00	0,00	0,00	0,00	0,00	0,00
0,00	4,03	0,07	0,50	0,53	1,00	0,03
0,00	0,03	0,36	0,00	0,03	3,00	2,03
4,00	0,07	0,03	0,39	0,68	0,00	0,00
1,00	1,00	2,00	0,03	0,75	0,00	0,00
1,00	2,00	2,03	0,03	0,42	0,00	0,00
0,00	0,18	2,03	0,07	0,14	0,00	0,00
3,00	1,36	1,00	1,36	0,53	2,00	0,07
2,00	2,07	0,36	0,07	0,13	1,00	0,03
5,00	0,00	2,00	0,00	0,36	0,00	0,00
0,00	1,00	0,00	1,00	0,57	2,00	0,03
0,00	1,00	1,36	1,36	0,00	1,00	0,00
0,00	2,14	1,00	1,00	0,71	2,00	0,18
0,00	3,00	2,00	2,00	0,39	1,00	0,03
2,00	2,07	0,39	2,00	0,78	2,00	0,03
0,00	1,00	1,00	0,00	0,00	4,00	0,00
0,00	0,00	0,36	0,00	0,00	0,00	0,00
0,00	1,00	2,00	0,39	0,39	1,00	0,00
1,00	1,03	1,14	1,03	0,24	0,00	0,00
0,00	0,36	0,00	0,00	0,00	0,00	0,00
0,00	0,00	0,00	0,00	0,00	0,00	0,00
0,00	1,18	2,00	0,00	0,18	0,00	0,00
0,00	4,00	1,00	0,36	0,00	0,00	0,00
0,00	2,36	0,00	1,14	0,00	0,00	0,00
2,00	2,14	0,39	0,14	0,78	1,00	0,03
2,00	3,03	2,00	0,36	0,32	1,00	0,00
0,00	4,00	1,00	4,00	2,00	1,00	0,00
1,00	2,00	1,00	1,14	0,18	1,00	0,00
0,00	6,00	1,00	1,03	0,10	1,00	0,10
1,00	0,39	0,14	0,00	2,03	2,00	0,03
0,00	0,36	0,71	0,71	0,71	2,00	0,03
3,00	0,00	2,00	0,36	1,03	0,00	0,00

3,00	0,03	1,14	1,36	1,32	5,00	0,21
3,00	0,00	2,03	0,03	0,57	3,00	0,03
2,00	2,07	2,00	0,14	0,13	1,00	0,10
2,00	2,03	2,00	0,50	0,10	2,00	0,03
0,00	4,00	2,00	0,00	0,14	0,00	0,00
1,00	0,71	0,14	0,18	0,46	4,00	0,14
0,00	2,00	2,00	0,14	0,00	1,00	0,29
0,00	1,14	1,14	2,00	0,00	2,00	0,36
0,00	1,00	1,00	0,00	0,00	2,00	0,43
0,00	2,00	1,00	0,00	0,07	0,00	0,00
1,00	1,50	0,03	0,18	0,10	1,00	0,07
0,00	1,71	1,03	0,18	0,07	0,00	0,07
0,00	0,00	0,00	0,00	0,00	0,00	0,29
0,00	5,00	2,00	0,03	0,10	0,00	0,00
0,00	2,14	2,00	0,50	0,03	0,00	0,00
2,00	0,00	0,03	0,00	0,42	1,00	0,36
2,00	0,36	1,14	0,36	0,14	2,00	0,03
2,00	4,00	2,00	1,00	0,78	2,00	0,00
0,00	0,14	1,00	0,00	0,00	2,00	0,00
1,00	3,14	2,00	0,18	0,18	2,00	0,00
1,00	2,14	2,00	0,14	0,29	3,00	0,36
0,00	1,00	0,00	0,00	0,00	0,00	0,00
0,00	0,71	0,36	1,36	2,00	0,00	0,00
0,00	0,00	2,00	0,00	4,00	3,00	0,03
1,00	0,03	0,07	0,36	0,32	3,00	0,00
0,00	0,29	2,00	0,18	0,18	0,00	0,03
0,00	1,07	1,00	0,36	0,14	0,00	0,00
2,00	3,14	2,00	1,00	0,57	4,00	0,10
2,00	1,00	1,03	2,00	0,21	5,00	0,00

Softdrinks/Sparkling Fluids	Estimated Energy Intake
2,00	1732,41
2,00	2396,91
0,00	1627,01
0,00	2396,91
2,00	3461,40
0,03	1374,79
0,00	1827,87
0,00	2112,57
0,00	2874,32
0,00	3195,83
0,00	1349,65
0,00	1442,16
0,00	1833,43
2,00	2743,27
0,00	1290,09
0,03	2242,06
0,03	1770,77
2,00	2573,00
0,43	2223,94
0,00	2244,60
0,03	1459,00
0,00	2821,53
0,03	1761,90
0,00	2196,81
2,00	1642,13
2,00	2400,95
0,00	1434,34
0,00	1908,61
0,43	2900,01
0,00	1844,16
0,00	3615,53
0,43	1842,83
0,29	2879,32
0,00	1394,72
0,00	2438,20
0,00	1928,40
0,00	1358,45
0,00	1536,36
0,03	2874,32
0,03	2909,29
0,00	3615,53
0,00	1936,64
0,00	2277,85
0,43	1502,66
0,43	5656,75
2,00	1974,57

0,03	4063,44
2,00	3478,79
0,00	1818,39
0,43	2729,75
0,00	1305,07
2,00	2335,85
0,29	2126,77
0,00	1677,81
0,00	1927,86
0,00	1388,46
0,29	1850,28
0,00	2185,59
0,29	1842,83
0,00	1827,87
0,00	1613,66
0,00	1882,45
0,43	2438,20
1,00	3643,58
0,00	3110,77
2,00	2635,31
0,29	3110,77
0,00	1194,06
0,00	2571,95
0,00	2715,49
0,00	2921,76
0,29	1807,66
0,00	1384,87
1,00	3999,97
2,00	4569,41
