# Supplementary material for: Dietary and cardio-metabolic risk factors in patients with Obstructive Sleep Apnea: cross-sectional study
Source: PeerJ. 2017 Jun 21;5:e3259. doi: 10.7717/peerj.3259 (PMC5482261; doi:10.7717/peerj.3259)
Supplement: Table S1 [file peerj-05-3259-s002.pdf]

Suppl Table 1. Energy estimation from Food Frequency Questionnaire

**OSA**[1]	**Estimate**	**Std Error**	**95% Confidence Limits**	
0*	2428.9	209.0	2019.2	2838.7
1*	2343.2	228.2	1895.1	2791.3
2*	2074.6	186.2	1709.3	2439.8
3*	2357.7	206.5	1953.0	2762.4

[1] Obstructive Sleep Apnea

* differentiated stages of Obstructive Sleep Apnea severity
