# Supplementary material for: Dietary and cardio-metabolic risk factors in patients with Obstructive Sleep Apnea: cross-sectional study
Source: PeerJ. 2017 Jun 21;5:e3259. doi: 10.7717/peerj.3259 (PMC5482261; doi:10.7717/peerj.3259)
Supplement: Table S2 [file peerj-05-3259-s003.pdf]

Suppl Table 2. Variance Information

Variable	Variance			DF	Relative Increase in Variance	Fraction Missing Information	Relative Efficiency
	Between	Within	Total				
Alcohol intake	0.328311	23.00126	23.33614	71.022	0.014559	0.014358	0.999713
Systolic Blood Pressure	8.03E-05	0.000189	0.000271	45.969	0.433475	0.304984	0.993937
Diastolic Blood Pressure	6.13E-05	0.000231	0.000294	53.907	0.27042	0.21431	0.995732
BMI[1]	0.074048	0.476908	0.552437	60.781	0.158371	0.137377	0.99726
LDL-C[2]	0.001485	0.015854	0.017369	65.127	0.095546	0.087497	0.998253
HDL-C[3]	0.000209	0.001819	0.002032	63.59	0.117275	0.105368	0.997897
TC[4]	0.001813	0.019759	0.021608	65.268	0.093574	0.08584	0.998286
Triglycerides (Ln transformed)	0.000364	0.003335	0.003706	64.013	0.111242	0.100473	0.997995
Fasting glucose	3.57E-05	0.000445	0.000481	66.115	0.081777	0.07581	0.998486
Glucose Tolerence Test	0.000687	0.002291	0.002992	51.981	0.305907	0.235958	0.995303
REM: R stage[5]	0.009547	0.013266	0.023004	36.081	0.734078	0.427497	0.991523
NREM: N1 stage[6]	0.004765	0.008198	0.013059	40.116	0.59292	0.375743	0.992541
NREM: N2 stage	0.004392	0.005064	0.009544	32.634	0.884576	0.474085	0.990607
NREM: N3 stage	1.908014	2.359223	4.305397	33.911	0.824922	0.456545	0.990952
Wachting	1.058259	2.683704	3.763128	47.319	0.402214	0.289225	0.994249
Vitamin A	0.001135	0.011432	0.012589	64.72	0.101255	0.092259	0.998158
Vitamin C	0.000306	0.013981	0.014294	70.455	0.022333	0.021864	0.999563
Central Apnea	0.008117	0.029954	0.038233	53.574	0.276411	0.218049	0.995658
Mixed Apnea	0.005386	0.02756	0.033054	58.127	0.199357	0.167158	0.996668

Obstructive Apnea	0.005557	0.038463	0.04413	61.52	0.147356	0.129017	0.997426
Snoring	0.004412	0.026294	0.030794	59.936	0.171164	0.146892	0.997071
Age	0.042668	1.809998	1.85352	70.33	0.024045	0.023502	0.99953

[1] BMI-Body Mass Index

[2] LDL-C –low-density lipoprotein cholesterol

[3]HDL-C – high-density lipoprotein cholesterol

[4]TC – total cholesterol

[5] REM – rapid eye movement expressed as percentage of total sleep

[6]NREM – no rapid eye movement
